# Cytomegalovirus reactivation is frequent in multiple myeloma patients treated with daratumumab‐based regimens

**DOI:** 10.1002/cam4.7402

**Published:** 2024-07-21

**Authors:** Danilo De Novellis, Raffaele Fontana, Bianca Serio, Emilia Vaccaro, Roberto Guariglia, Denise Morini, Michela Rizzo, Valentina Giudice, Carmine Selleri

**Affiliations:** ^1^ Hematology and Transplant Center University Hospital “San Giovanni di Dio e Ruggi d'Aragona” Salerno Italy; ^2^ Department of Medicine Surgery and Dentistry “Scuola Medica Salernitana”, University of Salerno Baronissi Italy; ^3^ Transfusion Medicine University Hospital “San Giovanni di Dio e Ruggi d'Aragona” Salerno Italy

**Keywords:** cytomegalovirus reactivation, daratumumab, infectious disease, multiple myeloma

## Abstract

**Background:**

Viral reactivations are frequent in hematologial patients due to their cancer‐related and drug‐induced immunosuppressive status. Daratumumab, an anti‐CD38 monoclonal antibody, is used for multiple myeloma (MM) treatment, and causes immunosuppression by targeting CD38‐expressing normal lymphocytes. In this single‐center two‐arm real‐life experience, we evaluated incidence of cytomegalovirus (CMV) reactivation in MM patients treated with daratumumab‐based regimens as first‐ or second‐line therapy.

**Methods:**

A total of 101 consecutive MM patients were included in this study and were divided into two cohorts: daratumumab and nondaratumumab‐based (control) regimens. Patients treated with >2 lines of therapies were excluded to reduce the confounding factor of multi‐treated cases. Primary endpoint was the CMV reactivation rate.

**Results:**

CMV reactivation rate was significantly higher in the daratumumab cohort compared to control group (33% vs. 4%; *p* < 0.001), also with higher CMV‐DNA levels (>1000 UI/mL in 12% of cases; *p* < 0.05). However, only one subject developed a CMV disease with severe pneumonia, while 12% of patients were successfully treated with preemptive therapy with valganciclovir. No subjects in the control cohort required anti‐CMV agents (*p* = 0.02).

**Conclusion:**

Our single‐center retrospective experience showed that daratumumab might significantly increase the risk of CMV reactivation in MM, while currently underestimated and related to morbility and mortality in MM patients under treatments. However, further validation on larger and prospective clinical trials are required.

## 1 INTRODUCTION

Multiple myeloma (MM) accounts for 10% of all hematological malignancies, and is characterized by proliferation and accumulation in the bone marrow (BM) of clonal CD38+ CD138++ plasma cells and by overproduction of monoclonal proteins (M‐protein) leading to diffuse organ damage.^1^, ^2^ Despite dramatic improvement in overall survival of MM in recent years due to the introduction of novel drugs, this disease remains incurable with an unfavorable prognosis related to a high relapse rate even after transplantation, and an increased infectious risk.^3^, ^4^, ^5^ Several mechanisms underlie this immune system deficiency, including disease‐induced immunosuppression, leukopenia, impaired T‐cell functions, steroid therapies, hypogammaglobulinemia, older age, severe renal dysfunction, poor performance status, and therapy‐related immunosuppresion.^6^, ^7^ The infectious risk is maximal during the first 3 months of therapy in all patients, decreasing in responding subjects and increasing in relapsed/refractory (RR) MM.^8^ Therefore, infectious complications are a frequent cause of morbidity and mortality in MM.^9^, ^10^

Daratumumab, a fully humanized IgGκ anti‐CD38 monoclonal antibody, exerts its anti‐MM activity through numerous mechanisms, such as antibody‐dependent cellular cytotoxicity, antibody‐dependent cellular phagocytosis, direct cellular apoptosis, and modulation of extracellular ectoenzyme activity.^4^ CD38, a surface membrane glycoprotein, is highly expressed on malignant plasma cells, and normal B, T, and natural killer lymphocytes; therefore, its pharmacologic targeting results in neoplastic cell killing and also in immune system impairment with increased infectious risk.^11^ In clinical trials, the overall infectious risk under daratumumab treatment is estimated ~38% across different studies, for both newly‐diagnosed and RR MM, with bacterial pneumonia as the most common infectious complication, leading to treatment discontinuation in 1%–4% of cases.^12^, ^13^ Daratumumab‐induced immunosuppression is also associated with an increased risk of viral reactivation, including Hepatitis B virus, Hepatitis C virus, and Herpes Zoster virus, leading to treatment delays, interruptions, or discontinuation.^12^

Cytomegalovirus (CMV) is a common pathogen with a prevalence of 83% in general population, and clinical manifestations vary from asymptomatic in healthy people to severe end‐organ dysfunction in immunocompromised subjects, including hematological patients and post‐transplant recipients.^14^, ^15^, ^16^ After primary CMV infection, immunocompentent subjects remain silent carriers for life, unless under certain conditions, such as immunosuppression, that causes viral reactivation. As for other viral reactivations, also for CMV, the reactivation risk might be increased by daratumumab treatment.^17^ However, to our knowledge, few data are available on the CMV reactivation rate in MM patients, with a lack of specific prophylaxis in current guidelines,^18^ and the association of viral reactivation with daratumumab therapies has not been clearly established yet. CD38 plays an important role in T cell‐mediated immune responses, as it facilitates the interaction between several molecules involved in immunological synapsis and the engagement of Natural Killer cells, T and B cells.^19^, ^20^ Indeed, CD38 can translocate to the immunological synapse after TCR engagement, and modulates antigen‐mediated T cell activation.^12^, ^19^, ^20^, ^21^ Therefore, anti‐CD38 blocking agents can impair normal immune responses against pathogens and increase the risk of infectious disease development.^12^, ^19^, ^20^, ^21^, ^22^

Because of CD38 roles in physiological T cell‐mediated immune responses against viral infections and reactivation,^22^ we sought to investigate the incidence of CMV reactivation and disease in MM patients treated with daratumumab‐based regimens as first‐ or second‐line therapy in a single‐center, two‐arm, real‐life experience.

## 2 PATIENTS AND METHODS

### 2.1 Study cohorts

A total of 101 consecutive MM patients from 2015 to November 2023 under active first‐ or second‐line treatment were included in this single‐center retrospective study, and were divided into two cohorts: daratumumab and nondaratumumab‐based (control group) regimens. Patients treated with >2 therapy lines were excluded to reduce confounding factors, such as advanced disease, very poor patient conditions, and deep immunosuppressive status.^23^ A control group was employed to investigate the impact of other factors involved in viral reactivation, including high‐dose glucocorticoids or the use of proteasome inhibitors.^24^, ^25^ Inclusion criteria were: age ≥ 18 years old; diagnosis of MM according to the International Myeloma Working Group (IMWG) criteria^26^; active first‐ or second‐line anti‐MM therapy outside clinical trials. This study was conducted in accordance with the Declaration of Helsinki, and protocols approved by our Ethics Committee “Campania Sud” (Brusciano, Naples, Italy; prot./SCCE n. 24,988). All patients provided written informed consent.

### 2.2 CMV quantification and serology

Lymphocyte count and anti‐CMV immunoglobulin G (IgG) levels were measured in patients at diagnosis and at reactivation. Hypogammaglobulinemia was defined as total IgG levels <400 mg/dL. Plasma CMV‐DNA copy number was quantified by real‐time TaqMan CMV‐DNA PCR according to manufacturers' instructions (Roche), monthly at every chemotherapy cycle, as per our institution's guidelines. After diagnosis of CMV reactivation, CMV‐DNA levels were assessed weekly until negativization. The instrument cut‐off for positive results was CMV‐DNA copy number > 137 copies/μL, as previously described.^16^ CMV reactivation was defined as CMV‐DNA copy number > 137 copies/μL in anti‐CMV IgG+ patients without organ damage, while CMV disease was defined when CMV‐related organ damage occurred, including pneumonia, gastrointestinal disease, hepatitis, retinitis, central nervous system disease, nephritis, myocarditis, and pancreatitis, associated to the detection of CMV‐DNA on the involved organ.^27^ Anti‐CMV agents were initiated when weekly progressively increasing in CMV‐DNA levels were observed.

### 2.3 Endpoints and statistical analysis

The primary endpoint was incidence of CMV reactivation with or without CMV‐related organ damage, and secondary endpoint was the incidence of CMV disease. Data were collected in spreadsheets and were analyzed using R statistical software (v. 4.0.5; RStudio) and SPSS (v. 25; IBM). Continuous variables were expressed as mean or median and compared with the Wilcoxon rank‐sum or student's *t*‐test. Categorical variables were expressed as counts and percentages and compared using Chi‐square or Fisher's exact test as appropriate. A *p* value of <0.05 was considered statistically significant.

## 3 RESULTS

### 3.1 Clinical characteristics at enrollment

A total of 101 consecutive MM patients were included in this study, with similar median age (66 years old vs. 67 years old, daratumumab vs. control group; *p* = 0.47) and sex ratio (M/F, 59%/41% vs. 58%/42%, daratumumab vs. control group; *p* = 0.9) between cohorts (Table 1). In control group, a slightly higher prevalence of micromolecular MM (*p* = 0.19) and κ type of involved free light‐chain (*p* = 0.17) was observed. Comorbidity frequencies were comparable between groups, including renal disfunction with a glomerular filtration rate (GFR) <40 mL/min (25% vs. 18%, daratumumab vs. control group; *p* = 0.39), rate of dialysis (4% vs. 6%, daratumumab vs. control group; *p* = 0.68), diabetes (18% vs. 20%, daratumumab vs. control group; *p* = 0.83), and increased body weight (median, 70 Kg vs. 70 Kg, daratumumab vs. control group; *p* = 0.56). Median follow‐up was 14 and 13 months in the daratumumab and control group, respectively (*p* = 0.5).

**TABLE 1:** Patients' characteristics at enrollment.

Characteristics	Daratumumab cohort *N* = 51	Control cohort *N* = 50	*p* Value
Median age, years (range)	66 (44–86)	67 (45–83)	0.47
Gender, *n* (%)			
Male	30 (59)	29 (58)	0.9
Female	21 (41)	21 (42)	
ECOG score, *n* (%)			
0–1	48 (95)	48 (96)	0.8
M‐protein type, *n* (%)			
IgG	39 (76)	37 (74)	
IgA	8 (16)	2 (4)	0.19
Micromolecular	3 (6)	11 (22)	
Not secernent	1 (2)	—	
Light chain type, *n* (%)			
Kappa	30 (59)	36 (72)	0.17
Lambda	21 (41)	14 (28)	
R‐ISS, *n* (%)			
I	12 (25)	11 (22)	0.7
II	20 (39)	22 (44)	
III	16 (32)	16 (32)	
Not available	3 (6)	1 (2)	
Extramedullary disease, *n* (%)	6 (12)	4 (8)	0.4
Median GFR, mL/min (range)	78 (4–117)	80 (4–118)	0.71
GFR < 40 mL/min, *n* (%)	12 (25)	9 (18)	0.39
Dialysis, *n* (%)	2 (4)	3 (6)	0.68
Diabetes, *n* (%)	9 (18)	10 (20)	0.83
Body weight, median, Kg (range)	70 (45–120)	70 (45–98)	0.56
Association regimens, *n* (%)			
Dara‐VTD	23 (45)		
Dara‐VMP	6 (12)		
Dara‐RD	11 (21)		
Dara‐VD	2 (12)		
Dara‐PD	1 (2)		—
Daratumumab single agent	4 (8)		
VRD		41 (82)	
KRD		7 (14)	
VMP		1 (2)	
KD		1 (2)	
Therapy setting, *n* (%)			
First line	37 (72)	42 (84)	
Second line	14 (28)	8 (16)	0.13
Prior ASCT	3 (6)	5 (10)	0.23

Abbreviations: dara‐PD, daratumumab‐pomalidomide‐dexamethasone; dara‐RD, daratumumab‐lenalidomide‐dexamethasone; dara‐VD, daratumumab‐bortezomib‐dexamethasone; dara‐VMP, daratumumab‐bortezomib‐melphalan‐prednisone; dara‐VTD, daratumumab‐bortezomib‐thalidomide‐dexamethasone; GFR, glomerular filtration rate; KD, carfilzomib‐dexamethasone; KRD, carfilzomib‐lenalidomide‐dexamethasone; R‐ISS, revised internatiolal staging system; VMP, bortezomib‐melphalan‐prednisone; VRD, bortezomib‐lenalidomide‐dexamethasone.

Therapeutic regimens employed in the daratumumab group were: daratumumab‐bortezomib‐thalidomide‐dexamethasone (dara‐VTD; *N* = 23; 45%); daratumumab‐lenalidomide‐dexamethasone (dara‐RD; *N* = 11; 21%); daratumumab‐bortezomib‐dexamethasone (dara‐VD; *N* = 6; 12%); daratumumab‐bortezomib‐melphalan‐prednisone (dara‐VMP; *N* = 6; 12%); daratumumab‐pomalidomide‐dexamethasone (dara‐PD; *N* = 1; 2%); and daratumumab as a single agent (*N* = 4; 8%). In the control group, regimens were: bortezomib‐lenalidomide‐dexamethasone (VRD; *N* = 41; 82%); carfilzomib‐lenalidomide‐dexamethasone (KRD; *N* = 7; 14%); VMP (*N* = 1; 2%); and carfilzomib‐ dexamethasone (KD; *N* = 1; 2%). Rates of enrolled patients at first‐ or second‐line therapy was similar in both groups (first‐line, 72% vs. 84%, daratumumab vs. control group; and second‐line, 28% vs. 16%, daratumumab vs. control group; *p* = 0.13), as weel as rates of previous autologous stem cell transplantation (6% vs. 10%, daratumumab vs. control group; *p* = 0.23).

### 3.2 CMV reactivation rate

Median lymphocyte count was similar in daratumumab and control cohorts (470 cells/μL vs. 480 cells/μL; *p* = 0.25), while median total serum IgG levels (346 mg/dL vs. 490 mg/dL; *p* = 0.39) and hypogammaglobulinemia (61% vs. 48%; *p* = 0.15) were slightly more represented in the daratumumab group compared to control subjects (Table 2).

**TABLE 2:** Cytomegalovirus (CMV) reactivation.

Characteristics	Daratumumab cohort *N* = 51	Control cohort *N* = 50	*p* Value
Lymphocyte count, median, ×10^3^/μL (range)	0.47 (0.1–1.8)	0.48 (0.2–1.19)	0.25
Antiviral prophylaxis with acyclovir, *n* (%)	32 (62)	43 (86)	**0.017**
Total IgG, median, mg/dL (range)	346 (37–2470)	490 (79–1150)	0.39
Total IgG < 400 mg/dL, *n* (%)	31 (61)	24 (48)	0.15
Anti‐CMV IgG at diagnosis, *n* (%)			
Yes	16 (31)	5 (10)	**0.03**
No	3 (6)	2 (4)	
Not available	32 (63)	43 (86)	
CMV reactivation, *n* (%)	17 (33)	2 (4)	**<0.001**
CMV DNA at peak, median UI/mL (range)	192 (34.5–141,000)	137 (137–137)	0.64
CMV DNA at peak >1000 UI/mL	6 (12)	0	**0.03**
Time to CMV reactivation, median, days (range)	29 (10–184)	54 (48–59)	0.64
Clinical features of CMV reactivation, *n* (%)			
Pneumonia	1 (2)	0	0.31
Blood reactivation	16 (31)	2 (4)	**<0.001**
Treatment with anti‐CMV agents	7 (14)	0	**0.02**

*Note:* *p* value < 0.05 was considered statistically significant (in bold).

Antiviral prophylaxis with acyclovir was less frequently employed in the daratumumab arm (62% vs. 86%; *p* = 0.02), while CMV reactivation rate was significantly higher in this cohort (33% vs. 4%; *p* = 0.001), with six patients (12%) showing a CMV‐DNA copy number > 1000 UI/mL (12% vs. 0%, daratumumab vs. control group; *p* = 0.03). Time to CMV reactivation was shorter in the daratumumab cohort, although this was not significant (29 vs. 54 days, daratumumab versus control group; *p* = 0.64). Moreover, the only subject who developed CMV disease was in the daratumumab arm, and the patient had severe grade III interstitial pneumonia associated with high CMV‐DNA levels (141.000 UI/mL) treated with intravenous ganciclovir. Among the remaining patients with CMV reactivation (6 out of 7), preemptive therapy with oral valganciclovir was successfully employed.

## 4 DISCUSSION

A high incidence of infections (overall transversal risk, 38%), especially upper respiratory tract infections and pneumonia, has been reported in several daratumumab trials,^13^ because of its immunosuppressive actions, including secondary hypogammaglobulinemia, depletion of regulatory B/T lymphocytes and natural killer (NK) cells, and neutropenia.^28^, ^29^, ^30^, ^31^, ^32^ Although the risk of bacterial infections and hepatitis virus or HZV reactivation has been clearly assessed, few data are available on CMV reactivation risk, without a clear recommendation for anti‐CMV prophylaxis.^13^, ^17^ In this single‐center, two‐arm, retrospective, real‐life study, we investigated the CMV reactivation rate in MM patients treated with the anti‐CD38 monoclonal antibody daratumumab as first‐ or second‐line therapy, and outcomes were compared to a daratumumab‐naïve control group.

To the best of our knowledge, our real‐life experience is the first study investigating CMV reactivation risk by comparing the results of daratumumab‐treated to daratumumab‐naïve MM patients in a fairly large number of nonmultirefractory subjects (<2 therapy lines; *N* = 51 and *N* = 50, respectively). Multi‐treated patients were excluded, as their very poor clinical conditions, uncontrolled disease, or several previous lines of immunosuppressive therapy might have been confounding factors, while other risk factors for immunosuppression, such as diabetes or dialysis, were balanced between groups.

CMV reactivation in daratumumab‐treated MM patients has only been anectodically reported with only single case in a heavily pretreated MM patient under single‐agent daratumumab therapy (7th line) and treated with intravenous ganciclovir 5 mg/kg twice a day, and with other three heavily pretreated MM cases of severe CMV‐related enterocolitis requiring prolonged anti‐CMV treatment.^33^, ^34^ In other previous studies, in a cohort of 13 RR MM patients (median of previous therapy lines, 5) under daratumumab‐containing regimens, the CMV reactivation rate has been assessed to 38%, while in a cohort of 15 MM patients with 3.5 median prior therapy lines, the reactivation rate has been reported at 73%, a very high incidence likely because 47% of these subjects have been previously received autologous stem cell transplantation.^18^, ^35^, ^36^ Conversely, in our study, rates of prior autologous stem cell transplantation were very low in both cohorts, without significant differences between groups. CMV reactivation/infection might also frequently co‐occur with Epstein–barr virus reactivation in MM patients under daratumumab treatment (17% of cases), especially in multi‐treated RR MM.^37^ Proteasome inhibitors can also increase the risk of CMV reactivation in plasma cell dyscrasias (MM or light chain amyloidosis)^25^; however, in our control group, almost the entire cohort received proteasome inhibitors (bortezomib, 84%; and carfilzomib, 16%) without influencing CMV reactivation risk, as the rate was much lower (4%) than that previously reported for proteasome inhibitor‐treated MM patients (39%).^25^ Daratumumab is given as 1 weekly infusion during cycles 1 to 2 (weeks 1 to 8), then as 1 infusion every 2 weeks (twice per 4‐week cycle; cycles 3 to 6; weeks 9 to 24), and subsequently as 1 infusion every 4 weeks (cycle 7+; week 25+ until disease progression), according to approved dosing schedule. In our daratumumab group, median time to CMV reactivation was 29 days, that was during weekly daratumumab administration, suggesting that closer dosing causes a more pronounced immunosuppression and increases the risk of viral reactivation, because of the important role of CD38 in T cell‐mediated immune responses.^22^

In our report, only one patient suffered CMV‐related pneumonia under dara‐VTD regimen; detection of CMV‐DNA in bronchoalveolar lavage was not performed because of suggestive radiological signs of interstitial pneumonia by lung CT scan, and a very high plasma viral load (141.000 UI/mL). Our patient was successfully treated with intravenous ganciclovir, and restarted anti‐MM treatment after pneumonia resolution. In all other cases with an increased viral load, preemptive therapy was promptly started to reduce the risk of CMV disease development.

Our study has several limitations: (i) retrospective nature of this investigation, although clinical characteristics were well‐balanced between groups; (ii) unavailability of anti‐CMV IgG serostatus at baseline for the entire study population, thus we can not exclude primary CMV infection rather than reactivations, although primary disease occurs in younger subjects, as over half of general adult population has been infected by age 40^38^; (iii) preemptive therapy was started based on clinical decision, considering various clinical and laboratory factors (e.g., lymphocyte count),^39^ and not on a specific CMV‐DNA copy number level; and (iv) acyclovir prophylaxis was more frequently performed in control group, even though all patients received low acyclovir doses (400 mg/twice daily or lower doses, according to renal function).

In conclusions, daratumumab can increase the risk of viral infections and reactivations, including CMV reactivation. In our retrospective single‐center two‐arm observational study, a marked increase in CMV reactivation was reported in daratumumab‐treated MM patients, that could be successfully reduced with a prompt initiation of preemptive therapy by preventing CMV‐related disease development. Indeed, CMV reactivation might silently occur and lead to cytopenias or interstitial pneumonias, thus a regular CMV‐DNA monitoring could both reduce antiviral prophylaxis‐related toxicity as therapy would be started as needed, and CMV disease development rates. MM patients treated with anti‐CD38 agents are at higher risk of infectious complications, especially pneumonia not Varicella‐Zoster Virus‐related, without an associated higher mortality rate.^12^ Other single‐center real‐life experiences or case series have reported that CMV reactivation can be frequent in anti‐CD38 agent treated MM subjects in up to 38% of cases^35^, ^36^, ^40^, ^41^; however, symptoms are commonly mild with fever, while CMV disease development is rare.^40^ Despite severe disease is unfrequently observed and CMV monitoring could be expensive, time consuming, and not easily applicable to routinely clinical practice, it has been shown that CMV reactivation negatively impact on clinical outcomes of MM patients, as dose reduction or drug discontinuation might be required until disease resolution.^40^ A close monitoring might early identify mild CMV‐related symptoms (e.g., cytopenias or diarrhea), that could be gone unrecognized or misattributed as drug‐induced toxicity, and could allow prompt initiation of anti‐viral agents before severe disease development. Moreover, CMV reactivation might be more frequent than already reported, because CMV monitoring is not commonly employed in clinical practice, while when performed, viral reactivation can be found in a high percentage of treated subjects, as reported from ongoing clinical trials with daratumumab in association with bispecific antibodies.^42^, ^43^ Therefore, CMV‐DNA level assessment before starting each chemotherapy cycle could be a valuable laboratory tool for monitoring viral reactivation risk in MM patients under daratumumab treatment. However, further validation on larger and prospective clinical trials are required.

## AUTHOR CONTRIBUTIONS

**Danilo De Novellis:** Conceptualization (equal); investigation (equal); methodology (equal); writing – original draft (equal). **Raffaele Fontana:** Investigation (equal). **Bianca Serio:** Investigation (equal). **Emilia Vaccaro:** Investigation (equal); methodology (equal). **Roberto Guariglia:** Investigation (equal). **Denise Morini:** Investigation (equal). **Michela Rizzo:** Investigation (equal). **Valentina Giudice:** Formal analysis (equal); investigation (equal); writing – original draft (equal). **Carmine Selleri:** Conceptualization (equal); project administration (lead); supervision (lead); writing – review and editing (lead).

## FUNDING INFORMATION

This study received no external funding.

## CONFLICT OF INTEREST STATEMENT

The authors declare no conflict of interest.

## ETHICS STATEMENT

Protocol approved by local ethic committee (Ethics Committee “Campania Sud”, Brusciano, Naples, Italy; prot./SCCE n. 24,988).

## INFORMED CONSENT

Patients received informed consent obtained in accordance with the Declaration of Helsinki (World Medical Association 2013) and protocols approved by local ethic committee (Ethics Committee “Campania Sud”, Brusciano, Naples, Italy; prot./SCCE n. 24,988).

## STATEMENTS

The authors declare that the material is original, has not been published before nor is under consideration in any journal.

## Data Availability

Data are available upon request by the authors.
